# Flame retardant and smoke-suppressant rigid polyurethane foam based on sodium alginate and aluminum diethylphosphite

**DOI:** 10.1080/15685551.2021.1879451

**Published:** 2021-01-31

**Authors:** Wei Zhang, Zidong Zhao, Yun Lei

**Affiliations:** a Department of safety engineering, School of Environmental and Chemical Engineering, Shenyang Ligong University, Shenyang, China; b Department of Mining Engineering and Metallurgical Engineering, Western Australian School of Mines, Curtin University, Kalgoorlie Australia; c Department of gas research, Shenyang Research Institute, China Coal Technology & Engineering Group Corp, Fushun, China; State Key Laboratory of Coal Mine Safety Technology, Fushun, China

**Keywords:** Rigid polyurethane foam, thermal properties, flame-retardant, sodium alginate

## Abstract

In order to improve the flame-retardant effect and thermal behaviour of rigid polyurethane foam (RPUF), the flame retardancy of sodium alginate (SA), aluminium diethyl phosphite (ADPO_2_) and expandable graphite (EG) were proposed. First, the structures of RPUF with or without flame retardancy were confirmed by scanning electron microscopy (SEM). Additionally, the combustion behaviours and thermal performance of the flame-retardant polyurethane were evaluated through thermogravimetric analysis (TGA), limiting oxygen index (LOI) tests, and UL-94 tests. Finally, the cone calorimeter results reveled the RPUF/5ADPO_2_/7.5SA/7.5EG exhibit excellent thermodynamic properties. The results of the heat release rate (HRR), total heat release (THR), total smoke production (TSP), and smoke production rate (SPR) could demonstrate the smoke-suppressant and flame-retardant of polyurethane. The system of RPUF/ADPO_2_/SA/EG showed excellent flame-retardant in polyurethane.

## 1. Introduction

Rigid polyurethane foam (RPUF) is a material with one of the best thermal insulation effects in the world. Due to its good thermal insulation performance, cold resistance, anti-corrosion, heat insulation, high strength, lightweight and other excellent characteristics, rigid polyurethane foam has become an indispensable material for heat insulation, water-proofing, leak stoppage, and for use in different industrial sectors, such as construction, transportation, the chemical industry, refrigeration, and power [1,2,12-14]. However, pristine RPUF had poor flame-retardant properties; in particular, there was a source of fire, RPUF burned smoothly, and the flame spread rapidly and generated a large amount of smoke [16-19]. Therefore, it is essential to study the flame retardancy of RPUF to protect the safety of people’s lives and property.

Lei Liu et al. used nano zirconium amino-tris-(methylenephosphonate) and expandable graphite to modify the flame retardant, mechanical and thermal insulating properties of rigid polyurethane foam [3]; Sheng Xu et al. have shown the synergistic flame-retarding effect of alginate and layered double hydroxides [4]. Zhang et al. have shown the flame retardant of ADPO_2_ and EG [5]; SA has been used by other researchers to the flame-retardant effect in other fields [6,7]. No studies have shown that the sodium alginate (SA)/aluminium diethylphosphite (ADPO_2_)/expandable graphite (EG) system could react with polyurethane foam to achieve a better flame-retardant effect. In the manuscript, the synergies gained from SA, ADPO_2_ and EG would exhibit a great smoke suppression and flame-retardant in polyurethane.

## 2. Materials and methods

Polyether polyol (hydroxyl number: 54.5–58.5 mg/g; viscosity: 7000 mpa·s/25°C) was purchased from Guangzhou Huixiang Chemical Industry Co., Ltd. (Guangdong, China). Diphenylmethane diisocyanate (MDI; technical pure grade) was purchased from Jinan Dada Chemical Industry Co., Ltd. (Shandong, China). Expandable graphite (particle size: 1000 mesh; carbon content: 99.996%) was supplied by Henan Liugong Graphite Co., Ltd. (Henan, China). Aluminium diethyl phosphite (density at 20°C: 1.35 g/m^3^; particle size: 20 ~ 40 μm) was supplied by Shanghai Suisi Chemical Technology Co., LTD. Sodium alginate (SA; white powder; pH: 6–8; granularity: 100 mesh; viscosity: 100–800 mpa·s) was purchased from Shandong Xiangyu Biotechnology Co., LTD. The stabilizer (technical pure grade) was supplied from Shanghai Baiang Chemical Technology Co., Ltd. (Shanghai, China) [8].

**Figure 1.**
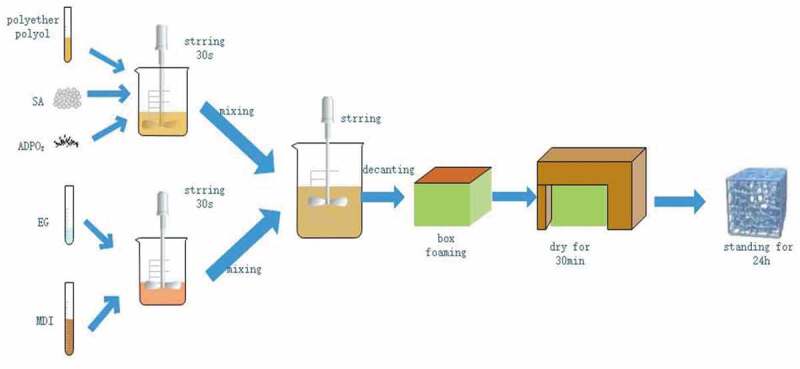
Production flow chart of RPUF/7.5SA/5ADPO_2_/5EG

All of the RPUF were prepared by box foaming (size: 100 mm×100 mm×10 mm). First, SA or ADPO_2_ was separately introduced into mould with RPUF, stirred for 30 s, held until foaming stabilization, then put into a drying oven, dried for 30 min at 80°C, and allowed to stand for 24 h at 25°C. Then, SA and ADPO_2_ were introduced to polyether polyol at the same time, stirred to mix well, and combined with MDI (with or without EG), stirred for 30 s, held until foaming stabilization in the box foaming, put into a drying oven, dried for 30 min at 80°C, and allowed to stand for 24 h at 25°C, as Figure 1. The combustion conditions of different samples were compared, and the ratio of SA to ADPO_2_ was adjusted (Table 1) to obtain the best flame-retardant effect.

**Table 1.** LOI values and UL-94 grades of different component samples

Samples	ADPO_2_/g	SA/g	EG/g	Polyether polyol/ml	MDI/ml	LOI/%	UL-94
PU-1	0	0	0	20	20	17.0	V-2
PU-2	6.25	6.25	0	20	20	20.5	V-1
PU-3	3.1	9.4	0	20	20	20.9	V-1
PU-4	5	7.5	0	20	20	21.5	V-1
PU-5	4.1	8.4	0	20	20	20.5	V-1
PU-6	12.5	0	0	20	20	20.0	V-2
PU-7	0	12.5	0	20	20	19.0	V-2
PU-8	5	7.5	2.5	20	20	22.0	V-0
PU-9	5	7.5	5	20	20	25.0	V-0
PU-10	5	7.5	7.5	20	20	25.5	V-0
PU-11	2.5	3.75	5	20	20	22.5	V-0
PU-12	0	7.5	5	20	20	19.0	V-2

A scanning electron microscope (SEM, FEI Nova Nano SEM 430) was used to observe the internal structure of the samples. Thermogravimetric analysis (TGA; STA8000 thermal gravimetric analyzer; Perkin Elmer) was used to measure the mass of the sample in relation to its temperature [9]. Each sample was measured under a nitrogen atmosphere with a 10°C/min rate from 0°C to 600°C. Samples with a size of 100 mm×100 mm×10 mm were prepared to test the Limit Oxygen Index (LOI) using a Fire Testing Technology (Germany Eager Technology Inc.), according to the ASTM D2863-19 standard. An FTT cone calorimeter was used to characterize the fire behaviour according to ISO 5660. The dimensions of the samples were 100 mm×100 mm×10 mm. The measured parameters included the heat release rate (HRR, kW/m^2^), total heat release (THR, MJ/m^2^), and total smoke release (TSR, m^2^/m^2^) [10].

## 3. Results and discussion

### 3.1 Morphology characterization

**Figure 2.**
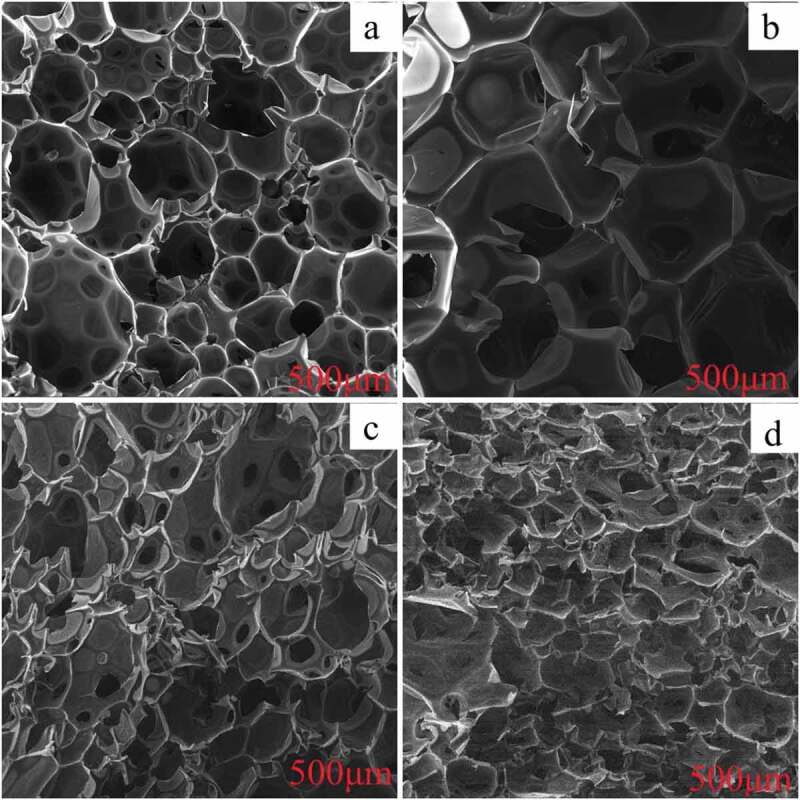
SEM images of various samples (a: pure RPUF; b: RPUF/7.5SA/5ADPO_2_; c: RPUF/7.5SA/5ADPO_2_/5EG; d: RPUF/7.5SA/5ADPO_2_/7.5EG)

To further explore the change in internal structure resulting from the addition of SA and ADPO_2_ to polyurethane, the internal structure of polyurethane was observed by scanning electron microscopy, as shown in Figure 2. Figure 2a shows the internal structure of the rigid polyurethane foam without flame retardant had large gaps. During burning, heat would be instantaneously transferred through these large gaps, resulting in an acceleration in burning speed. Apparently, the internal gap structure of RPUF with 7.5SA/5ADPO_2_ decreased than the pristine RPUF (Figure 2b). As shown in Figure 2b, when SA and ADPO_2_ were added to the mixed polyurethane, the internal structure was denser. With the amount of EG increasing, and the inner space of 7.5SA/5ADPO_2_/5EG became more compact than 7.5SA/5ADPO_2_. However, the SEM characterization of 7.5SA/5ADPO_2_/5EG (Figure 2c) was similar to 7.5SA/5ADPO_2_/7.5EG (Figure 2d). EG, as additive hard particles, would affect the bubble nucleation and bubble growth during the foaming process, further to damage the foam structure [11]. The addition of EG resulted in the collapse of large cell size and collapse cellular structure [8]. The system of SA/ADPO_2_/EG can have a synergistic effect that prevents further combustion after the reaction.

### 3.2 Flame retardancy

**Figure 3.**
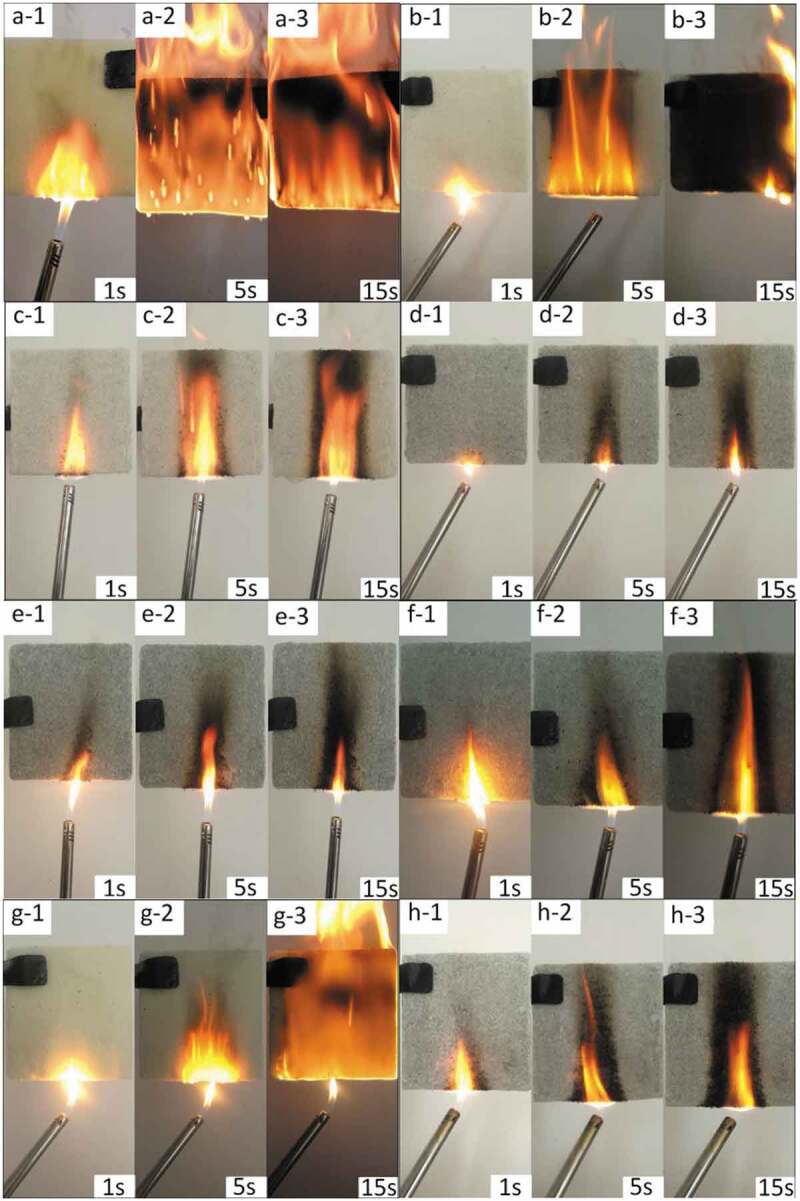
Combustion of different samples at different times (a:pure RPUF;b:RPUF/7.5SA/5ADPO_2_;c: RPUF/7.5SA/5ADPO_2_/2.5EG;d: RPUF/7.5SA/5ADPO_2_/5EG; e: RPUF/7.5SA/5ADPO_2_/7.5EG f: RPUF/3.75SA/2.5ADPO_2_/5EG;g: RPUF/7.5SA;h: RPUF/7.5SA/5EG)

The combustion state of the sample, heated at the bottom, was observed at 1, 5 and 15 s, as shown in Figure 3. When SA and ADPO_2_ were introduced into RPUF, the flame went out within 5S after the ignition source left, and the carbonization degree was reduced. LOI measuring instrument was determined the volume percentage of oxygen required during polymer combustion, and the flammability of the sample was tested by UL-94 vertical combustion tester. The results are shown in Table 1, and the standard deviation and standard error values were ±0.1%. The LOI of RPUF with SA (RPUF/12.5SA) increased from 17.0% (pristine RPUF) to 19.0%. The LOI value of RPUF with SA and ADPO_2_ (RPUF/5ADPO_2_/7.5SA) was 21.5%, higher than that of RPUF/6.25ADPO_2_/6.25SA and RPUF/3.1ADPO_2_/9.4SA.

According to the orthogonal method, the ratio of SA to ADPO_2_ was adjusted (Table 1). The combustion degree of the sample with a rate of SA to ADPO_2_ of 1:2 was less than 1:1. Upon adjusting the rate to 3:1 and 3:2, the latter rate had the best result. Based on these results, by adding EG, the flame-retardant effect was obviously increased, and the LOI of RPUF/5ADPO_2_/7.5SA/2.5EG, RPUF/5ADPO_2_/7.5SA/5EG and RPUF/5ADPO_2_/7.5SA/7.5EG was higher than those of RPUF/5ADPO_2_/7.5SA and pristine RPUF, and the UL-94 rating reached V0 without any dripping. However, compared with RPUF/5ADPO_2_/7.5SA/7.5EG, the flame-retardant effect of RPUF/5ADPO_2_/7.5SA/5EG was almost the same, and the increase in LOI was not apparent. Therefore, it was clear that at an ADPO_2_:SA:EG ratio of 2:3:3, the LOI value was the highest, and the UL-94 level was the highest.

**Figure 4.**
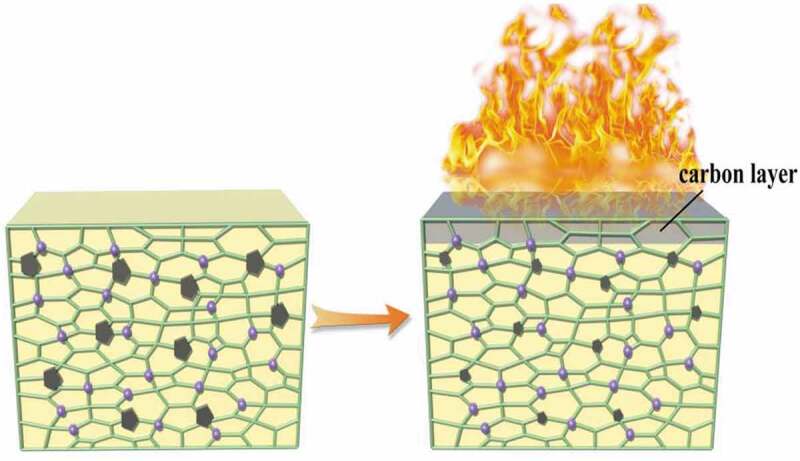
Combustion diagram of polyurethane internal network structure

The system of SA/ADPO_2_/EG has a synergistic flame-retardant effect. Although the flame-retardant effect of RPUF with SA (RPUF/12.5SA) was similar to that of RPUF with SA and EG (RPUF/7.5SA/5EG), the synergistic flame-retardant effect of only SA and EG was not obvious. When EG was introduced into RPUF with SA and ADPO_2_, the carbonized area of combustion was smaller, and the flame-retardant effect was much better than only adding SA and EG (Figure 3 g). When burned, the internal network structure of polyurethane was filled and shrunk, and there were many phosphorus elements distributed in the structure. When the sample was ignited, a layer of carbon layer formed on the surface, and the surface gap would be filled to prevent the further spread of flame to the interior as shown in Figure 4. The system of SA/ADPO_2_/EG could have an excellent flame-retardant effect on RPUF.

### 3.3 TGA tests

The Thermal decomposition behaviour of RPUF was analyzed by thermogravimetric analysis instrument [8]. The TGA data of the various samples are shown in Table 2. Below 250°C, the behaviours of the samples were nearly the same. At 220°C and 335°C, pure RPUF achieved T_5%_ and T_50%_.the synergistic effect of ADPO_2_ and SA made the RPUF/5ADPO_2_/7.5SA temperature of T_5%_ and T_50%_ delayed at about 240°C and 355°C. Over 300°C, pure RPUF decreased faster, only about 20% of the weight was left at 600°C, while RPUF/5ADPO_2_/7.5SA/5EG and RPUF/5ADPO_2_/7.5SA/7.5EG obviously showed that this system of SA/ADPO_2_/EG had a superior effect, and its weight loss rate was about 42%. The residue weight of RPUF/7.5SA/5EG with the same amount of EG was significantly lower than that of RPUF/5ADPO_2_/7.5SA/5EG. The residue weight of RPUF/5ADPO_2_/7.5SA/2.5EG was about 31% and about 42% of RPUF/5ADPO_2_/7.5SA/5EG. The residue weight of RPUF/2.5ADPO_2_/3.75SA/5EG was a little higher than RPUF/5ADPO_2_/7.5SA and RPUF7.5SA/5EG, which mean the system of SA/ADPO_2_/EG was better than RPUF with two kinds of them. This behaviour can be analyzed from the perspective that the treated RPUF has a composite structure composed of the hard cell structure and soft regions [8].

**Table 2.** TGA data of the various samples

Samples	T_5%_ (°C)	T_50%_ (°C)	Residue (wt%)
Pure RPUF	220	335	19.15
RPUF/5ADPO_2_/7.5SA	240	355	23.56
RPUF/5ADPO_2_/7.5SA/2.5EG	255	359	32.12
RPUF/5ADPO_2_/7.5SA/5EG	265	366	42.78
RPUF/5ADPO_2_/7.5SA/7.5EG	272	378	44.85
RPUF/2.5ADPO_2_/3.75SA/5EG	250	370	30.64
RPUF/12.5SA	228	342	21.23
RPUF/7.5SA/EG	235	347	25.56

### 3.4 Cone calorimeter

**Figure 5.**
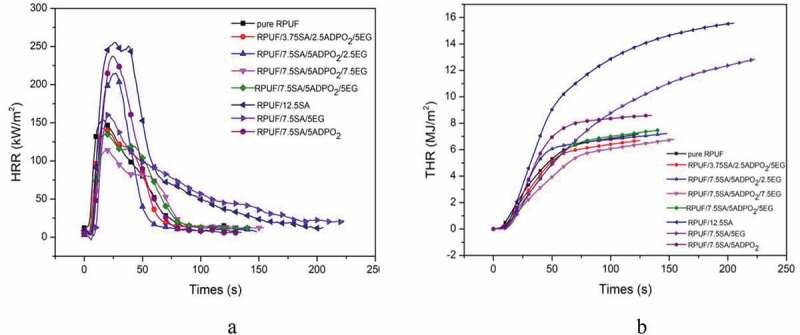
HRR and THR curves of different RPUF foam (a: HRR; b: THR;)

HRR, THR, SPR and TSP were explored to evaluate the smoke and fire risk of the materials [3]. From Figure 5, the combustion speed of the pristine RPUF was very fast, until the whole area was carbonized, it would stop burning. The peak heat release rate (PHRR) was 155 kW/m^2^, while the PHRR of RPUF/5ADPO_2_/7.5SA/7.5EG decreased by 22.6%. The THR value of RPUF/5ADPO_2_/7.5SA/7.5EG was approximately 6 MJ/m^2^. For SPR, RPUF/5ADPO_2_/7.5SA/7.5EG yielded the lowest value, while the TSP of RPUF/5ADPO_2_/7.5SA/7.5EG and RPUF/7.5SA/5EG was lower than that of RPUF. The PHRR of RPUF/5ADPO_2_/7.5SA/5EG and RPUF/2.5ADPO_2_/3.75SA/5EG could achieve 125 and 140 kW/m^2^; however, the PHRR of the sample without EG or little EG was higher than pure RPUF. ADPO_2_ and SA could not contribute to the decrease of HRR and THR. The synergistic effect of ADPO_2_/SA/EG could decrease the PHRR. Considered the charring ability of RPUF/5ADPO_2_/7.5SA/5EG, the residue weight was the highest. It can explain that the char residues formed during the combustion and prevent the transfer of heat and oxygen [15,20,21]. Additionally, the peak SPR of RPUF/5ADPO_2_/7.5SA/5EG was 0.06 m^2^, the lowest of all. The peak SPR of RPUF/5ADPO_2_/7.5SA/5EG and pure RPUF was similar. The TSP of RPUF/5ADPO_2_/7.5SA/5EG was lower than pure RPUF before the combustion time of 40 s; however, after 40 s, the TSP of RPUF/5ADPO_2_/7.5SA/5EG was higher than pure RPUF. The TSP of RPUF/7.5SA/5EG was the lowest. SA and EG contributed to the decrease in smoke. ADPO_2_ mainly contributed flame retardancy via promoting the formation of phosphorus-containing compounds in gas, leading to more smoke release [8]. During combustion, EG plays an important role in the flame retardant and smoke suppression. However, the synergistic flame retardant and smoke suppression effect of ADPO_2_/SA/EG was the bestFigure 6.

**Figure 6.**
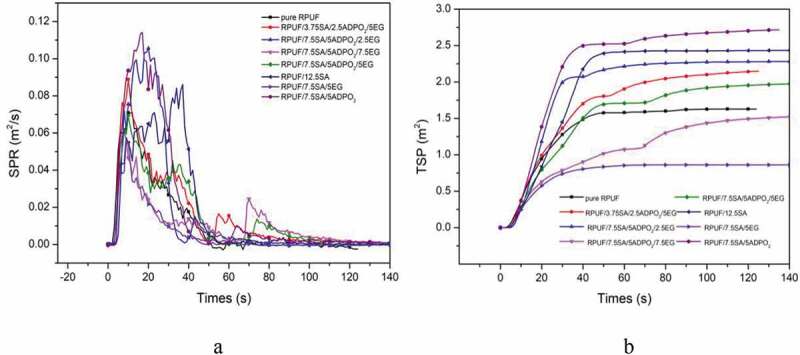
SPR and TSP curves of different RPUF foam (a: SPR; b: TSP)

## 4. Conclusions

When the flame retardancy of ADPO_2_/SA/EG was introduced into RPUF, a layer of carbon layer formed, and the net structure was changed [3]. The system of RPUF/7.5ADPO_2_/5SA/7.5EG showed excellent flame-retardant properties and smoke suppression compared with pristine RPUF, which decreased the value of PHRR to 125 kw/m^2^ and the THR was about 6MJ/m^2^, much lower than pure RPUF. The TSP of RPUF/7.5ADPO_2_/5SA/7.5EG was 1.5 m^2^/m^2^, while the TSP of RPUF/7.5SA/5EG was the lowest (0.8 m^2^/m^2^), which exhibit excellent smoke suppression. The LOI value of RPUF/7.5SA/5EG increased to 25.5% and the level of it could reach V0 by UL-94 test. The system of RPUF/ADPO_2_/SA/EG commonly used as insulation material in buildings showed excellent thermal properties, which could play an excellent role in the field of flame-retardant.
